# Genomic landscape and clinical characteristics of *ERBB2/HER2*-mutated colorectal cancers

**DOI:** 10.1093/oncolo/oyag302

**Published:** 2026-07-31

**Authors:** Muhammed Dogukan Aksu, Paola Zinser Peniche, Doga Kahramangil Baytar, Bhaghyasree Jambunathan, Yu Jen Alexander Jan, Ronan Hsieh, Shafia Rahman, John C Krauss, Christine M Veenstra, Ibrahim Halil Sahin

**Affiliations:** Division of Medical Oncology, Department of Oncology, Mayo Clinic, Rochester, MN, 55905, United States; Department of Medicine, Division of Hematology and Oncology, University of Pittsburgh School of Medicine, Pittsburgh, PA, 15213, United States; Department of Medicine, Division of Hematology and Oncology, University of Pittsburgh School of Medicine, Pittsburgh, PA, 15213, United States; Department of Medicine, Division of Hematology and Oncology, University of Pittsburgh School of Medicine, Pittsburgh, PA, 15213, United States; Department of Medicine, Division of Hematology and Oncology, University of Pittsburgh School of Medicine, Pittsburgh, PA, 15213, United States; Division of Hematology and Oncology, Swedish Cancer Institute, Seattle, WA, 98104, United States; Department of Internal Medicine, Division of Medical Oncology, The Ohio State University Comprehensive Cancer Center, Columbus, OH, 432120, United States; Division of Hematology and Oncology, University of Michigan Medical School, Ann Arbor, MI, 48109, United States; Division of Hematology and Oncology, University of Michigan Medical School, Ann Arbor, MI, 48109, United States; Division of Hematology and Oncology, University of Michigan Medical School, Ann Arbor, MI, 48109, United States

**Keywords:** colorectal neoplasms, colon, rectum, mutation, genomics, ERBB2/HER2

## Abstract

**Background:**

Molecular underpinnings and clinical characteristics of *ERBB2/HER2*-mutated colorectal cancers (CRCs) remain poorly understood. We aimed to comprehensively characterize the genomic landscape and clinical features of *ERBB2/HER2*-mutated CRC and to compare them with those of *ERBB2/HER2-*amplified CRC.

**Patients and methods:**

We analyzed cBioPortal data from 4348 CRC patients with available *ERBB2/HER2* profiling. Clinicodemographic features, microsatellite instability (MSI), tumor mutational burden, and alterations in 94 cancer-associated genes were evaluated using chi-square/Fisher’s exact test, Kruskal–Wallis test, and hierarchical clustering.

**Results:**

*ERBB2/HER2* putative driver mutations were identified in 95 patients (2.2%), amplifications in 87 (2.0%), and wild-type status in 4166 (95.8%). In the overall cohort, *ERBB2/HER2*-mutated tumors demonstrated significant *KRAS* enrichment (50.5%) compared to wild-type (37.7%; *P *= .014) and amplified tumors (18.4%; *P *< .001), alongside MSI enrichment (37.2% vs 1.6%; *P *< .001), elevated tumor mutational burden, and right-sided predominance. *ERBB2/HER2*-mutated tumors also showed broad driver co-alterations largely reflecting their MSI background. In contrast, *ERBB2/HER2*-amplified tumors exhibited left-sided predominance, with microsatellite stable (MSS)-restricted analyses revealing selective mutual exclusivity with *KRAS* (Log_2_ OR = −1.58; *q *= 0.019) and *TP53* co-occurrence (Log_2_ OR = 2.04; *q *= 0.019). Hierarchical clustering confirmed that mutated and amplified tumors consistently occupy separate clusters across anatomical sites.

**Conclusion:**

*ERBB2/HER2* mutations and amplifications represent molecularly and clinically distinct subgroups. *ERBB2/HER2*-mutated tumors are characterized by an MSI-enriched, hypermutated background with frequent concurrent *KRAS* alterations, whereas amplified tumors exhibit selective *KRAS* mutual exclusivity and *TP53* co-occurrence. These exploratory findings support alteration-specific therapeutic strategies in CRC.

Implications for Practice This large-scale genomic analysis reveals that *ERBB2/HER2*-mutated and *ERBB2/HER2*-amplified colorectal cancers represent distinct molecular and clinical entities that might require different therapeutic approaches. *ERBB2/HER2*-mutated tumors frequently arise on an MSI-enriched, hypermutated background with concurrent *KRAS* alterations, suggesting a need for combination therapies and heightened resistance potential. Conversely, *ERBB2/HER2*-amplified tumors show *KRAS* mutual exclusivity and *TP53* co-occurrence on a chromosomally unstable background, making them more suitable for focused HER2-targeted therapy. These findings reinforce the critical importance of molecular stratification beyond simple HER2 positivity, suggesting alteration-specific treatment algorithms and informing clinical trial design for precision oncology approaches in colorectal cancer.

## Introduction

Colorectal cancer (CRC) represents the third most common cancer and the second most common cause of cancer-related deaths globally; the 5-year survival rate for metastatic CRC remains dismal at less than 20%.^1^^,^^2^

The molecular underpinnings of CRC are considered to be a key determinant of clinical behavior and therapeutic response. The most commonly followed molecular classification categorizes CRCs by 3 major genomic instability patterns: microsatellite instability (MSI), the CpG island methylator phenotype (CIMP), and chromosomal instability.^3^ The Consensus Molecular Subtypes (CMS) classification further identifies 4 distinct subtypes: CMS1 (microsatellite instability immune, 14%), hypermutated, microsatellite unstable and strong immune activation; CMS2 (canonical, 37%), epithelial, marked WNT and MYC signaling activation; CMS3 (metabolic, 13%), epithelial and evident metabolic dysregulation; and CMS4 (mesenchymal, 23%), prominent TGF-β signaling and stromal infiltration.^4^ Next-generation sequencing, however, has revealed even greater complexity, identifying several molecularly distinct subgroups in which specific driver mutations influence tumor biology and treatment sensitivity.^5^ For example, *BRAF* mutations carry significant biological heterogeneity and they have distinct impacts on disease biology, treatment response, and clinical outcomes.^6^

Among the targetable oncogenic drivers in CRC, human epidermal growth factor receptor 2 (*ERBB2*, also known as *HER2*) represents a particularly compelling therapeutic opportunity.^7^^,^^8^ ERBB2/HER2 is a member of the epidermal growth factor receptor (EGFR) family and plays crucial roles in cell proliferation, survival, and migration by activating downstream PI3K/AKT and RAS/RAF/MEK pathways.^9^ In CRC, *ERBB2/HER2* alterations occur through 2 distinct mechanisms: gene amplification and mutations.^9^  *ERBB2/HER2* amplifications in CRC have been associated with resistance to anti-EGFR therapies and generally poor prognosis.^10^ However, therapeutic advances have transformed the management of patients with *ERBB2/HER2*-amplified CRC.^11^^,^^12^

In contrast, *ERBB2/HER2* mutations in CRC remain far less well-characterized despite representing a similarly prevalent alteration. Unlike the relatively uniform mechanism of *ERBB2/HER2* amplification, *ERBB2/HER2* mutations encompass a diverse spectrum, with point mutations detectable in up to 3% of CRC patients.^13^ Preclinical studies suggest that different *ERBB2/HER2* mutations may have distinct functional consequences and therapeutic sensitivities, with some showing resistance to trastuzumab but sensitivity to tyrosine kinase inhibitors.^14^ However, clinical data on *ERBB2/HER2*-mutated CRC remain limited to small case series and retrospective analyses. Furthermore, it remains unclear whether *ERBB2/HER2*-mutated and *ERBB2/HER2*-amplified CRCs represent distinct molecular subtypes with different co-occurring genomic alterations, pathway dependencies, and clinical characteristics.

To address these knowledge gaps, we conducted a comprehensive genomic analysis using a large, multi-institutional cohort from the cBioPortal database. In this study, we aimed to characterize the clinical, demographic, and genomic features of *ERBB2/HER2*-mutated CRC compared to *ERBB2/HER2*-amplified and wild-type disease to inform precision medicine approaches.

## Patients and methods

**Figure 1.**
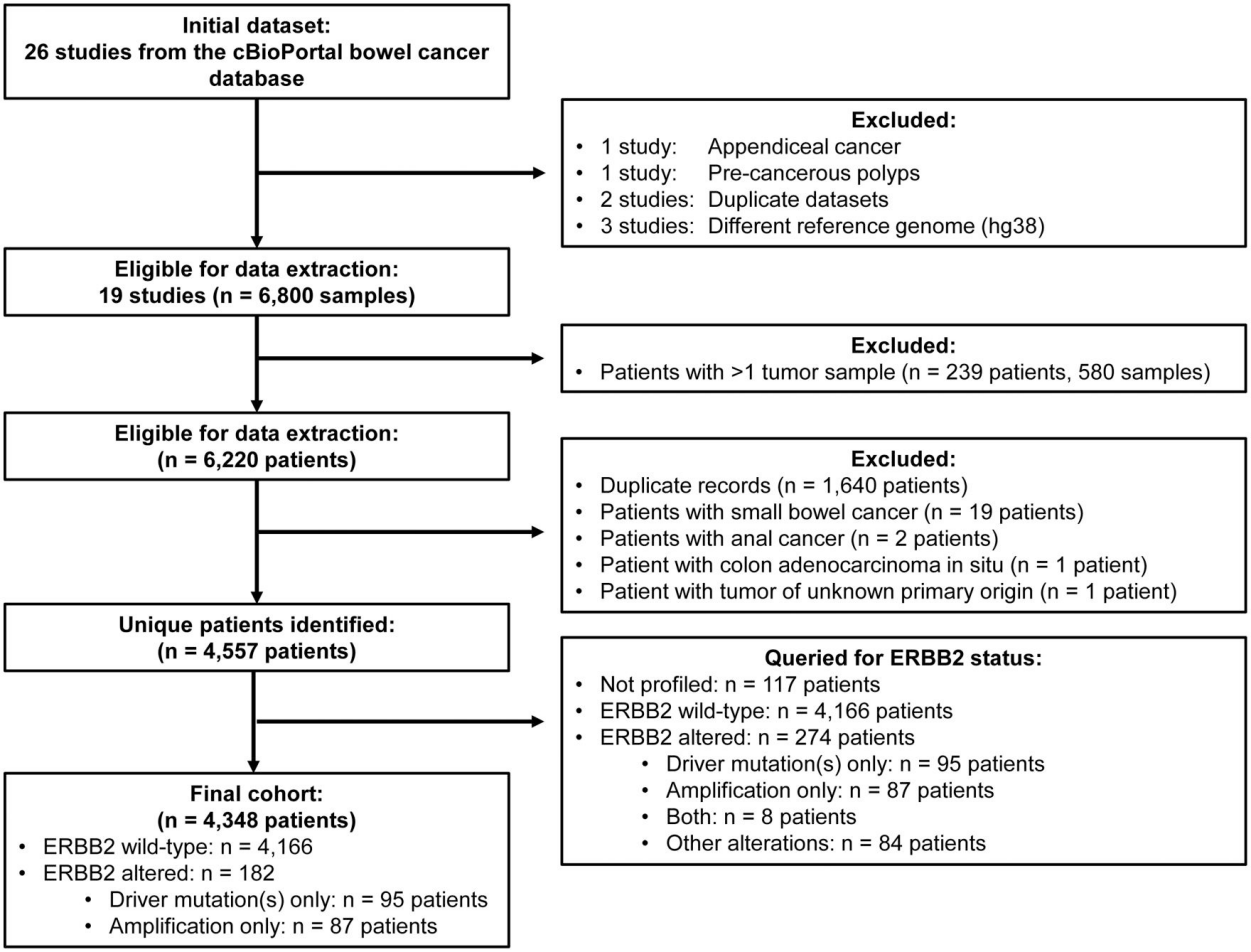
Study flowchart and patient selection criteria. The cBioPortal bowel cancer database was queried, yielding 26 initial studies. After excluding studies involving appendiceal cancer, pre-cancerous polyps, duplicate datasets, and those using a different reference genome (hg38), 19 studies comprising 6,800 samples were eligible for data extraction. Patients were subsequently excluded if they had multiple tumor samples (*n* = 239 patients, 580 samples), duplicate records (*n* = 1640), or non-colorectal primary sites, including small bowel cancer (*n* = 19), anal cancer (*n* = 2), colon adenocarcinoma in situ (*n* = 1), or tumor of unknown primary origin (*n* = 1), resulting in 4557 unique patients. Following the *ERBB2/HER2* query, patients without profiling of mutation and copy number data (*n* = 117) and patients with concurrent *ERBB2/HER2* driver mutations and amplifications (*n* = 8) or other alterations without driver events (*n* = 84) were excluded to ensure valid comparative analyses. The final analytic cohort comprised 4348 patients: 4166 *ERBB2/HER2*-wild-type, 95 with *ERBB2/HER2* putative driver mutations only, and 87 with *ERBB2/HER2* amplifications only.

The cBioPortal for Cancer Genomics (https://www.cbioportal.org) is a comprehensive web-based platform that aggregates genomic and clinical data from major cancer sequencing studies.^15^ In this study, the cBioPortal for Cancer Genomics was systematically queried to identify patients with CRC and available *ERBB2/HER2* profiling. Of the 26 studies listed under the bowel cancer category, 19 met eligibility criteria for further curation and analysis (Figure 1).^16–32^ After removing patients with multiple samples, duplicate entries, and non-colorectal primaries, a total of 4,557 unique patients remained, each with a single sample. These patients were subsequently queried for *ERBB2/HER2* alteration status. Copy number alteration data were derived from either putative copy-number alterations from Genomic Identification of Significant Targets in Cancer (GISTIC) 2.0 analysis^33^ or copy number calls from targeted OncoPanel sequencing, depending on the contributing study. *ERBB2/HER2* amplification was defined as high-level copy number gain (GISTIC score +2 or equivalent OncoPanel call), as annotated within cBioPortal and consistent with standard genomic profiling conventions used across the contributing studies. *ERBB2/HER2* putative driver mutations were defined based on OncoKB annotation,^34^ which represents the standard variant classification framework integrated within cBioPortal, classifying variants as oncogenic or likely oncogenic based on functional and clinical evidence. Among the cohort, 274 patients harbored at least one *ERBB2/HER2* alteration, while 117 patients had no profiling data available for both mutation analysis and copy number variation detection. To maximize statistical power and minimize selection bias, patients with either a known *ERBB2/HER2* mutation or amplification were retained in the analysis. In the final cohort, patients were further excluded if they exhibited both driver mutation(s) and amplifications concurrently. For patients harboring both amplifications and mutations of unknown significance, classification was based on the presence of the driver alteration (amplification), and these cases were included in the amplification group (*n* = 4 patients with this pattern). *ERBB2/HER2* wild-type patients were defined as those without any genetic alterations in *ERBB2/HER2*, regardless of predicted pathogenicity. The final study cohort included 4,348 patients, consisting of 95.8% (*n* = 4,166) *ERBB2/HER2* wild-type cases, 2.2% (*n* = 95) cases with *ERBB2/HER2* putative driver mutations only, and 2.0% (*n* = 87) cases with *ERBB2/HER2* amplifications only.

Patients’ demographic and clinical variables, along with molecular features, were extracted directly from cBioPortal. All entries were systematically reviewed for data consistency and quality. In cases where conflicting or inconsistent data were identified, the affected observations were excluded from subsequent analyses to maintain data integrity. The total number of complete and valid observations for each variable is reported in Table 1.

**Table 1.** Baseline clinical and genomic characteristics of patients with *ERBB2/HER2*-mutated, *ERBB2/HER2*-amplified, and *ERBB2/HER2* wild-type colorectal cancer.

	*ERBB2/HER2* mutated (*n* = 95)	*ERBB2/HER2* amplified (*n* = 87)	*ERBB2/HER2* wild-type (*n* = 4166)	*P* value
**Female, *n* (%)^a^**	36 (40.0%)	30 (34.9%)	1954 (48.2%)	.017
**Age (years), mean ± SD^b^**	58.7 ± 15.0	54.4 ± 14.1	58.9 ± 14.8	.013
**Race, *n* (%)^c^**				.098
** White**	42 (71.2%)	55 (78.6%)	1824 (80.1%)	
** African American**	8 (13.6%)	5 (7.1%)	262 (11.5%)	
** Asian**	8 (13.6%)	8 (11.4%)	170 (7.5%)	
** Other**	1 (1.7%)	2 (2.9%)	21 (0.9%)	
**Cancer type, *n* (%)^d^**				.107
** Colon cancer**	50 (56.2%)	53 (63.1%)	2668 (66.5%)	
** Rectal cancer**	39 (43.8%)	31 (36.9%)	1347 (33.5%)	
**Tumor site, *n* (%)^e^**				.005
** Left colon**	19 (38.8%)	36 (70.6%)	1297 (51.1%)	
** Right colon**	30 (61.2%)	15 (29.4%)	1242 (48.9%)	
**Microsatellite instability, *n* (%)^f^**	29 (37.2%)	1 (1.6%)	284 (9.6%)	<.001
**Tumor mutational burden, mean ± SD^g^**	33.8 ± 44.8	6.1 ± 7.6	10.9 ± 20.9	<.001
**Total mutation count, mean ± SD^h^**	237.5 ± 935.2	15.4 ± 28.2	128.1 ± 427.5	<.001
**Fraction of genome altered, mean ± SD^i^**	0.14 ± 0.17	0.23 ± 0.16	0.20 ± 0.16	<.001

Patient characteristics were compared across 3 groups: *ERBB2/HER2*-mutated, *ERBB2/HER2*-amplified, and *ERBB2/HER2*-wild-type. Superscript letters indicate the number of patients with available data for each variable, in the respective order:

a Gender: *n* = 90, 86, and 4054.

b Age: *n* = 90, 87, and 3948.

c Race: *n* = 59, 70, and 2277.

d Cancer type: *n* = 89, 84, and 4015.

e Tumor site (excluding rectal cancer): *n* = 49, 51, and 2539.

f Microsatellite instability: *n* = 78, 64, and 2944.

g Tumor mutational burden: *n* = 95, 77, and 3774.

h Total mutation count: *n* = 95, 76, and 3764.

i Fraction of genome altered: *n* = 71, 85, and 3211. Statistical analyses were performed using the chi-square test for categorical variables, the Fisher exact test for race, and the Kruskal–Wallis test for continuous variables. *P* values < .05 were considered statistically significant.

To contextualize *ERBB2/HER2* alterations within the broader oncogenic landscape of CRC, a total of 94 genes were manually curated from core and extended oncogenic pathways based on established roles in CRC biology as defined in prior large-scale genomic studies and published pathway literature.^28^^,^^35–39^ These genes encompass tumorigenesis (CRC core and WNT, RAS-RAF-MAPK, TGF-β/SMAD, BMP/SMAD), therapeutic targeting (PI3K-AKT-mTOR, receptor tyrosine kinases, cell cycle, and growth control), and immune evasion (immune regulation and antigen presentation): CRC core and WNT (*APC*, *ARID1A*, *AXIN2*, *FAT3*, *FAT4*, *FBXW7*, *GSK3B*, *RNF43*, *SOX9*, *TCF7L2*, *TP53*); RAS-RAF-MAPK (*ARAF*, *BRAF*, *HRAS*, *KRAS*, *MAP2K1*, *MAP2K2*, *MAPK1*, *MAPK3*, *MRAS*, *NRAS*, *RAF1*, *RRAS*); PI3K-AKT-mTOR (*AKT1*, *AKT2*, *AKT3*, *INPP4B*, *MTOR*, *PIK3CA*, *PIK3CB*, *PIK3CD*, *PIK3R1*, *PTEN*, *RICTOR*, *RPTOR*); TGF-β/SMAD (*SMAD2*, *SMAD3*, *SMAD4*, *SMAD7*, *TGFB1*, *TGFB2*, *TGFB3*, *TGFBR1*, *TGFBR2*, *TGFBR3*, *TGFBRAP1*); BMP/SMAD branch (*BMP1*, *BMP10*, *BMP15*, *BMP2K*, *BMP3*, *BMP4*, *BMP5*, *BMP6*, *BMP7*, *BMP8A*, *BMP8B*, *BMPER*, *BMPR1A*, *BMPR1B*, *BMPR2*, *SMAD1*, *SMAD5*, *SMAD6*, *SMAD9*, *TGFB1I1*, *TGFBI*); receptor tyrosine kinases (*EGFR*, *ERBB3*, *ERBB4*, *FGFR1*, *FGFR2*, *FGFR3*, *IGF1R*, *KIT*, *MET*, *PDGFRA*); immune regulation and antigen presentation (*B2M*, *CD274*, *CTLA4*, *HLA-A*, *HLA-B*, *HLA-C*, *LAG3*, *PDCD1*, *TAP1*, *TAP2*); and cell cycle & growth control (*CCNE1*, *CDK4*, *CDK6*, *CDKN2A*, *MYC*, *RB1*, *STK11*). This gene panel was then queried across the final cohort of 4348 patients with CRC to assess pathway-level alteration patterns and visualize genomic landscapes. Specifically, for Figure 3 and Figure S3, a focused subset of 10 genes was selected for comparative driver alteration analysis based on their established role as the most frequently altered driver genes in CRC and their particular relevance as downstream convergence pathways of *ERBB2/HER2* signaling, where enrichment patterns are most likely to carry biological and therapeutic significance.^28^^,^^35^^,^^40^^,^^41^

### Statistical analysis

All statistical analyses were performed using R (version 4.4.1; R Foundation for Statistical Computing). Categorical variables were analyzed using the chi-square test or Fisher’s exact test, depending on the expected counts. Results were summarized as numbers and frequencies. Continuous variables were compared using the Kruskal–Wallis test and reported as mean ± SD. For comparative analysis of driver alteration frequencies across *ERBB2/HER2*-defined subgroups, an overall Fisher’s exact test was first performed across all 3 groups for each gene; pairwise comparisons were subsequently conducted when the overall test was significant, with Benjamini–Hochberg correction applied across all pairwise tests within each gene to control the false discovery rate (FDR; *q* values). Unsupervised hierarchical clustering was performed on a gene alteration frequency matrix representing the percentage of patients with each alteration across *ERBB2/HER2*-defined subgroups, computed separately for colon and rectal cancers. Euclidean distance and complete linkage were used for both row and column clustering, implemented via the “ComplexHeatmap” package in R. Associations between *ERBB2/HER2* status and pathogenic gene alterations were evaluated using odds ratios (OR) from contingency tables, with Fisher’s exact test for significance testing and FDR correction as described above. Given that cBioPortal aggregates data from studies using panels with variable gene coverage, alteration frequencies for each gene were calculated using only patients with confirmed profiling data for that gene as the denominator. Similarly, co-occurrence and mutual exclusivity analyses were restricted to patients with profiling data available for both genes under comparison. All visualizations were created in R using “ggplot2,” “ComplexHeatmap,” “dplyr,” and related packages. Throughout the analyses, *P-*values < .05 were considered statistically significant, and *q* values < 0.05 (FDR-corrected) were considered significant for multiple comparison analyses. The specific statistical tests, along with corresponding *P* and *q* values where applicable, are reported in the figure legends.

## Results

### Patient demographics and genomic characteristics by ERBB2/HER2 status

Baseline clinical and genomic characteristics are summarized in Table 1. The frequency of *ERBB2/HER2* putative driver mutations was 2.2% (*n* = 95), *ERBB2/HER2* amplification was 2.0% (*n* = 87), and *ERBB2/HER2* wild-type was 95.8% (*n* = 4166).

Among the clinical and demographic characteristics, race and cancer type (colon vs rectal) were found to be comparable across the *ERBB2/HER2*-mutated, *ERBB2/HER2*-amplified, and wild-type groups (*P *= .098 and *P *= .107, respectively). Age distribution differed significantly among the groups, with patients harboring *ERBB2/HER2* amplification being younger (54.4 ± 14.1 years) compared to those with mutations (58.7 ± 15.0 years) and wild-type genotype (58.9 ± 14.8 years; *P *= .013). A significant sex distribution difference was also observed, with wild-type tumors being more frequent in female patients (48.2%) compared to *ERBB2/HER2*-mutated (40.0%) or amplified tumors (34.9%; *P *= .017). Tumor anatomical distribution in colon cancers differed significantly by *ERBB2/HER2* status (*P* = .005), with *ERBB2/HER2*-mutated tumors more frequently in the right colon (61.2% vs 48.9% in wild-type) and amplified tumors in the left colon (70.6% vs 51.1%). MSI was present in 37.2% of *ERBB2/HER2*-mutated tumors and 9.6% of wild-type tumors, whereas there was only one case (1.6%) with an MSI tumor in the *ERBB2/HER2*-amplified cohort (*P *< .001). Given that MSI tumors inherently harbor increased mutational burden, we evaluated TMB among microsatellite stable (MSS) tumors only (*n* = 49, *n* = 63, and *n* = 2,660 for *ERBB2/HER2*-mutated, amplified, and wild-type, respectively). *ERBB2/HER2*-mutated tumors demonstrated elevated TMB compared to both amplified and wild-type groups (7.7 ± 3.9 vs 5.4 ± 2.5 and 6.8 ± 12.6, respectively; *P *< .001). In the overall cohort, *ERBB2/HER2*-mutated tumors similarly showed higher TMB (33.8 ± 44.8 vs 6.1 ± 7.6 and 10.9 ± 20.9; *P *< .001). Of note, TMB values were derived from cBioPortal’s pre-computed calculations without exclusion of the defining *ERBB2/HER2* variant; however, a single point mutation contributes negligibly to genome-wide TMB. Regarding chromosomal instability, while *ERBB2/HER2*-amplified tumors showed a higher fraction of genome altered in the overall cohort (0.23 ± 0.16 vs 0.14 ± 0.17 and 0.20 ± 0.16; *P *< .001), this difference was not observed when restricting to MSS tumors (0.22 ± 0.19 vs 0.20 ± 0.17 and 0.20 ± 0.16; *P *= .914).

#### Genomic landscape and pathway alterations across ERBB2/HER2 subgroups

**Figure 2.**
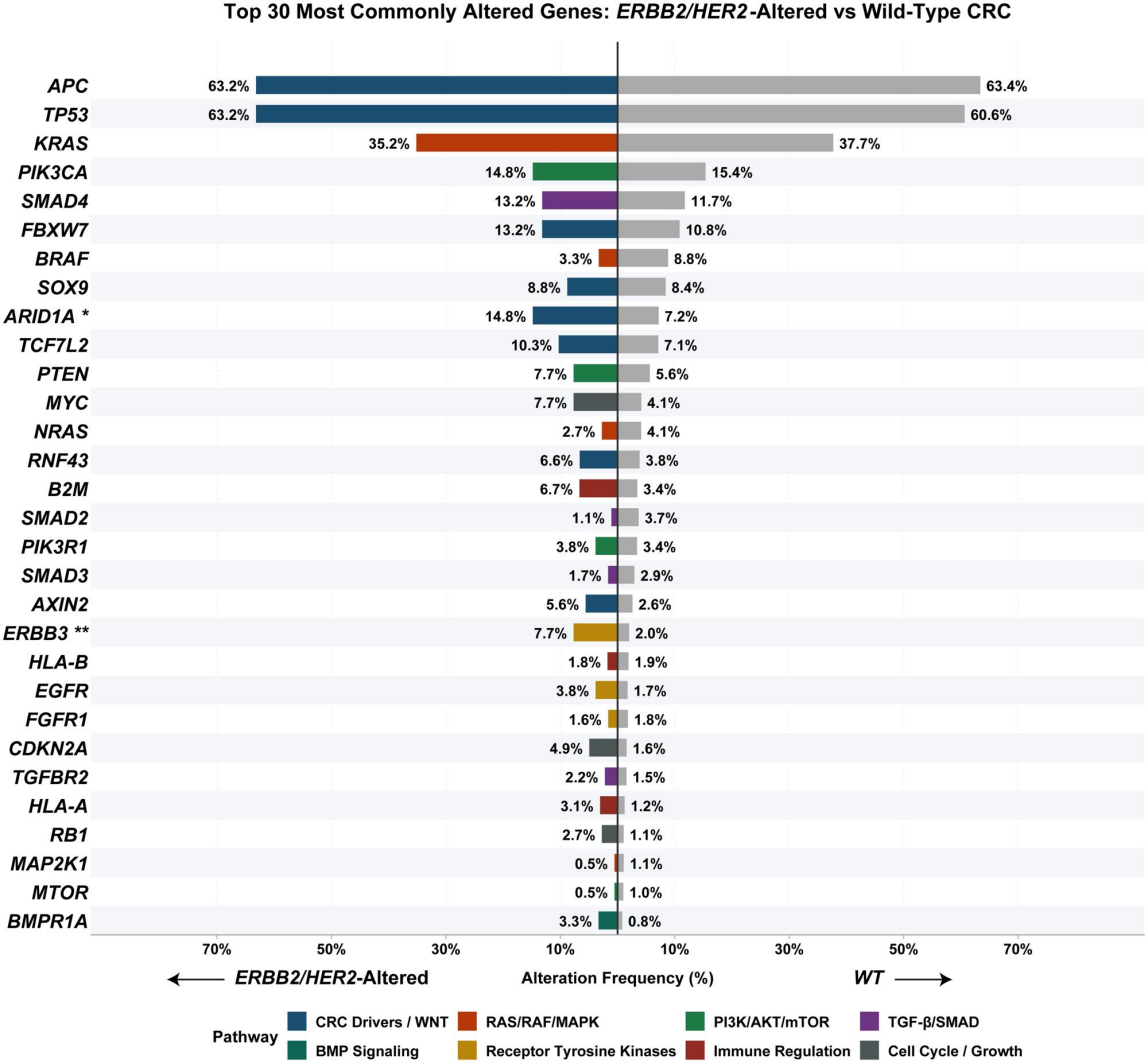
Comparative driver genomic alteration frequencies of the 30 most commonly altered genes between *ERBB2/HER2*-altered and wild-type colorectal cancer. Alteration frequencies for the top 30 most commonly altered genes across the overall cohort (*n* = 4348) are shown for *ERBB2/HER2*-altered (*n* = 182) and wild-type (*n* = 4166) subgroups. Genes are ranked by descending overall alteration frequency and color-coded by oncogenic pathway. Patients without profiling data for a given gene were excluded from its frequency calculation. Statistical comparisons were performed using Fisher’s exact test with Benjamini-Hochberg false discovery rate correction. Significance levels indicate adjusted *P* values annotated on gene labels: *P < .05, **P < .01.

Comparative alteration frequencies between *ERBB2/HER2*-altered and wild-type tumors across the 30 most commonly altered genes in the overall cohort are illustrated in Figure 2. Canonical CRC drivers dominated the alteration landscape across both groups, with *APC* (63.2% vs 63.4%), *TP53* (63.2% vs 60.6%), and *KRAS* (35.2% vs 37.7%) altered at broadly comparable frequencies between *ERBB2/HER2*-altered and wild-type tumors, respectively. Beyond these top drivers, *PIK3CA* (14.8% vs 15.4%) and *SMAD4* (13.2% vs 11.7%) showed similarly comparable rates across the 2 groups. Notably, *ERBB3* (7.7% vs 2.0%; *q *< 0.01) and *ARID1A* (14.8% vs 7.2%; *q *< 0.05) were significantly enriched in *ERBB2/HER2*-altered tumors. The comprehensive genomic alteration frequencies across all genes for the overall cohort and the MSS-restricted cohort are provided in Figures S1 and S2, respectively. These revealed broadly consistent alteration frequencies across pathways, with *APC* (74.7%), *TP53* (74.2%), *KRAS* (43.4%), *PIK3CA* (16.4%), and *SMAD4* (15.8%) maintaining similar relative dominance in MSS-restricted patients.

**Figure 3.**
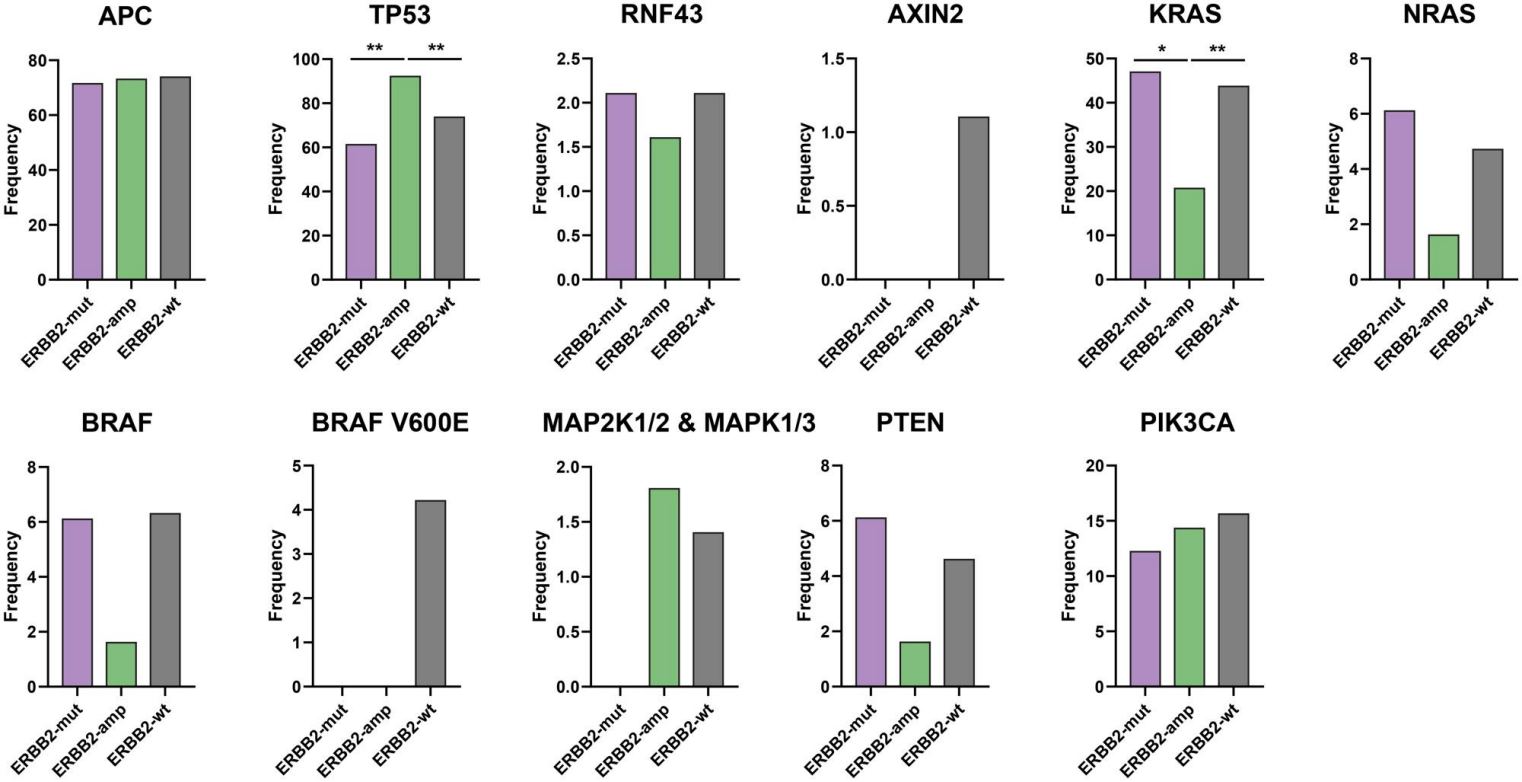
Comparative analysis of driver gene alterations across *ERBB2/HER2*-defined colorectal cancer subgroups among microsatellite stable (MSS) patients. Putative driver alteration frequencies for selected key genes are shown across *ERBB2/HER2*-mutated (*n* = 49), *ERBB2/HER2*-amplified (*n* = 63), and *ERBB2/HER2* wild-type (*n* = 2660 ) subgroups. Genes are organized by pathway: CRC core/WNT signaling (*APC*, *TP53*, *RNF43*, *AXIN2*), RAS/RAF/MAPK signaling (*KRAS*, *BRAF* (all alterations), *BRAF* V600E, *NRAS*, *MAP2K1/2* & *MAPK1/3*), and PI3K/AKT/mTOR signaling (*PTEN*, *PIK3CA*). Statistical comparisons were performed using Fisher’s exact test for overall group differences across all 3 subgroups, followed by pairwise comparisons with Benjamini–Hochberg false discovery rate correction applied across all pairwise tests. Significance levels indicate Benjamini–Hochberg-adjusted *P* values: *P < .05, **P < .01.

Comparative analysis of driver alterations of selected genes across *ERBB2/HER2* subgroups restricted to MSS tumors is presented in Figure 3, with the overall cohort analysis provided in Figure S3. *APC*, the most frequently altered gene in CRC, showed similar mutation frequencies across MSS subgroups (71.4%, 73.0%, and 73.8% for mutated, amplified, and wild-type tumors, respectively; *P *> .05), as well as in the overall cohort (64.2%, 62.1%, and 63.4%, respectively; *P *> .05). Within the RAS/RAF/MAPK pathway, *ERBB2/HER2*-mutated MSS tumors demonstrated the highest *KRAS* alteration frequency (46.9%), followed by wild-type (43.7%) and amplified tumors (20.6%), with *ERBB2/HER2*-amplified tumors showing significantly reduced *KRAS* alterations compared to both mutated (*P *= .031) and wild-type groups (*P *= .004), consistent with the overall cohort analysis (mutated: 50.5%, wild-type: 37.7%, amplified: 18.4%; mutated vs amplified *P *< .001, amplified vs wild-type *P *< .001, mutated vs wild-type *P *= .014). *BRAF* driver alterations were less frequent in amplified tumors compared to mutated and wild-type tumors in both the MSS-restricted (1.6% vs 6.1% and 6.3%, respectively; *P *> .05) and overall cohort analyses (1.1% vs 5.3% and 8.8%, respectively; *P *= .018 for amplified vs wild-type). To further characterize these *BRAF* alterations, we separately examined *BRAF* V600E driver alterations across subgroups. In the overall cohort, *BRAF* V600E was present in 1.1% of *ERBB2/HER2*-mutated, 0% of amplified, and 7.2% of wild-type tumors; the depletion in *ERBB2/HER2*-amplified tumors relative to wild-type was statistically significant (*q *= 0.008). In the MSS-restricted analysis, *BRAF* V600E was absent in both *ERBB2/HER2*-mutated and amplified MSS tumors (0% each) compared to 4.2% in wild-type. *TP53* alterations were significantly more prevalent in *ERBB2/HER2*-amplified MSS tumors (92.1%) compared to both mutated (61.2%; *P *= .004) and wild-type cases (73.6%; *P *= .004), consistent with the overall cohort (amplified: 77.0%, mutated: 50.5%, wild-type: 60.6%; amplified vs mutated *P *< .001, amplified vs wild-type *P *= .003). *PIK3CA* alterations were comparable across all MSS subgroups (mutated: 12.2%, amplified: 14.3%, wild-type: 15.6%; all *P *> .05) and the overall cohort (all *P *> .05). No significant differences were observed for the remaining genes presented in Figure 3, including *RNF43*, *AXIN2*, *NRAS*, *MAP2K1/2* and *MAPK1/3*, and *PTEN*, in the MSS-restricted analysis.

#### Anatomical site-specific genomic patterns and co-occurrence analysis

To evaluate whether tumor anatomical site influences the genomic similarity patterns of *ERBB2/HER2*-defined subgroups, we performed unsupervised hierarchical clustering based on subgroup-level gene alteration frequencies for both MSS-restricted tumors (Figure S4) and the overall cohort (Figure S5), understanding that this approach is descriptive in nature and, by design, precludes *de novo* group discovery. In MSS colon cancers, *ERBB2/HER2*-mutated tumors formed a distinct cluster separate from both wild-type and amplified groups, with wild-type and amplified tumors clustering together, consistent with the overall cohort analysis. In MSS rectal cancers, *ERBB2/HER2*-mutated and wild-type tumors clustered more closely, while amplified tumors formed a separate cluster. Importantly, across both anatomical sites in both MSS-restricted and overall cohort analyses, *ERBB2/HER2*-mutated and amplified tumors occupied distinct clusters. However, transcriptomic analyses will be further needed to validate expression level differences and characterize these subgroup distinctions.

**Figure 4.**
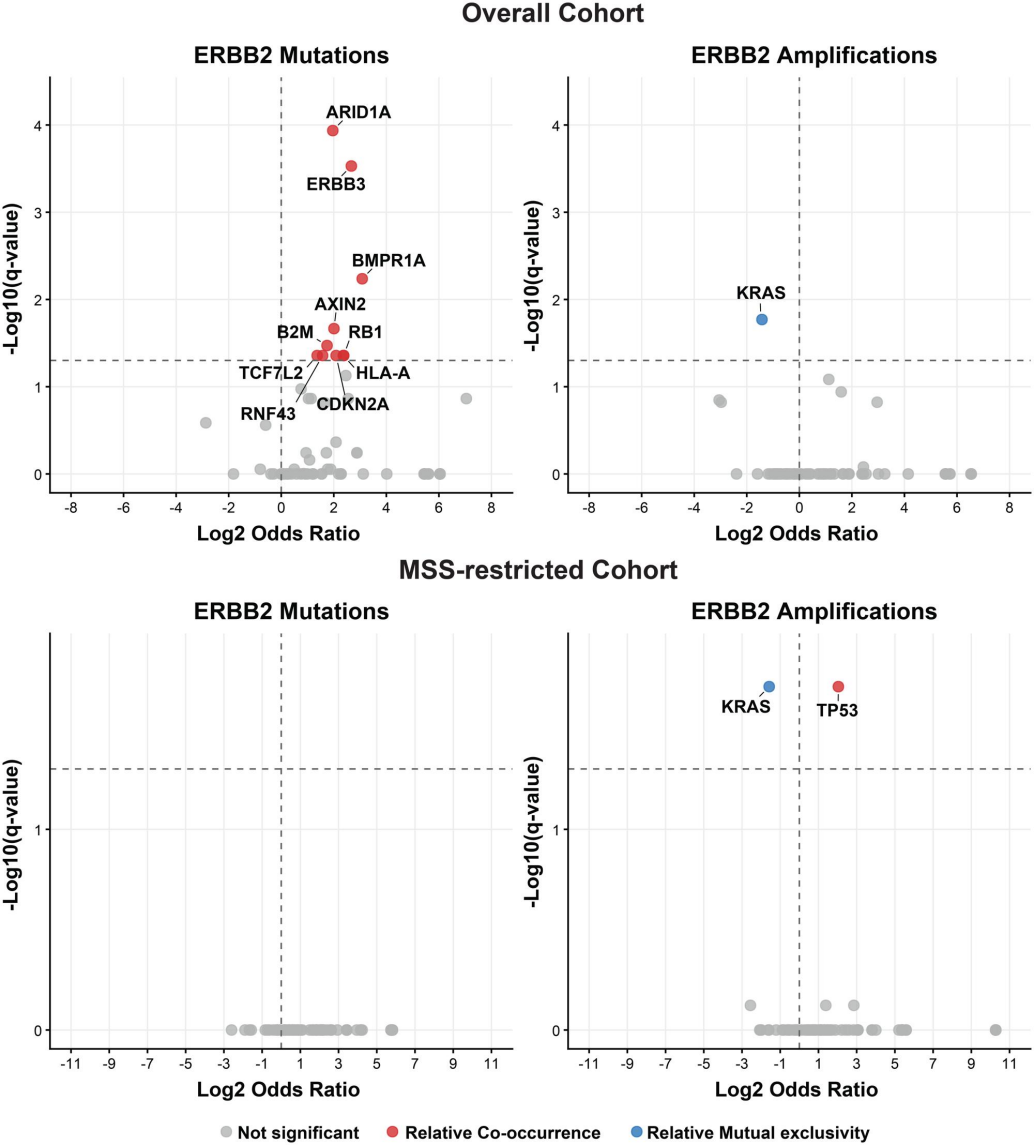
Associations of *ERBB2/HER2* alterations with selected oncogenic gene alterations. Volcano plots displaying statistical associations between *ERBB2/HER2* mutations or amplifications and putative driver alterations across the curated 94-gene panel in the overall cohort and in the microsatellite stable (MSS)-restricted cohort. In the overall cohort, sample sizes were *n* = 95 for *ERBB2/HER2*-mutated and *n* = 87 for *ERBB2/HER2*-amplified tumors; in the MSS-restricted analysis, *n* = 49 and *n* = 63, respectively. Each point represents a gene, with color coding indicating association patterns: red denotes relative co-occurrence (*q *< 0.05, Log_2_ OR > 0), blue indicates relative mutual exclusivity (*q *< 0.05, Log_2_ OR < 0), and gray represents non-significant associations. The horizontal dashed line marks the significance threshold (*q *= 0.05), and the vertical dashed line indicates a neutral odds ratio (Log_2_ OR = 0). Statistical analyses employed Fisher’s exact test with Benjamini–Hochberg correction for false discovery rate control.

We next assessed co-occurrence and mutual exclusivity patterns between *ERBB2/HER2* alterations and the 94-gene panel in both the overall cohort and MSS-restricted tumors (Figure 4). In the overall cohort analysis, the *ERBB2/HER2*-mutated subgroup demonstrated significant co-occurrence with ten genes: *ARID1A* (Log_2_ OR = 1.96, *q *< 0.001), *ERBB3* (Log_2_ OR = 2.67, *q *< 0.001), *BMPR1A* (Log_2_ OR = 3.08, *q *= 0.006), *AXIN2* (Log_2_ OR = 2.01, *q *= 0.022), *B2M* (Log_2_ OR = 1.74, *q *= 0.034), *RNF43* (Log_2_ OR = 1.57, *q *= 0.044), *RB1* (Log_2_ OR = 2.38, *q *= 0.044), *TCF7L2* (Log_2_ OR = 1.37, *q *= 0.044), *HLA-A* (Log_2_ OR = 2.36, *q *= 0.044), and *CDKN2A* (Log_2_ OR = 2.09, *q *= 0.044). Among *ERBB2/HER2*-amplified tumors, significant mutual exclusivity with *KRAS* was observed (Log_2_ OR = −1.42, *q *= 0.017), while *TP53* co-occurrence showed a consistent directional effect (Log_2_ OR = 1.12, *q *= 0.082). In the MSS-restricted analysis, *ERBB2/HER2*-amplified tumors retained selective mutual exclusivity with *KRAS* (Log_2_ OR = −1.58, *q *= 0.019) and significant co-occurrence with *TP53* (Log_2_ OR = 2.04, *q *= 0.019), while no significant associations were identified for the *ERBB2/HER2*-mutated subgroup. This likely reflects the reduced statistical power of the smaller MSS subgroup (*n* = 49 for mutated) and the known enrichment of several co-altered genes, including *ARID1A*,^42^  *B2M*,^43^  *HLA-A*,^44^  *RNF43*,^45^  *TCF7L2*,^46^  *ERBB3*,^47^ and *AXIN2*,^46^ in MSI tumors.

## Discussion

To our knowledge, this comprehensive genomic analysis of 4,348 colorectal cancer patients represents one of the largest systematic characterizations of *ERBB2/HER2* alterations in CRC. Our findings demonstrate that while *ERBB2/HER2* alterations collectively affect approximately 4.2% of CRC patients, mutations (2.2%) and amplifications (2.0%) exhibit fundamentally different biological behaviors.

*ERBB2/HER2*-mutated tumors arise within a hypermutated, MSI-enriched genomic context and frequently harbor concurrent *KRAS* driver alterations. In the overall cohort, those tumors show co-occurrence with ten genes spanning chromatin remodeling (*ARID1A*), WNT signaling (*RNF43*, *AXIN2*, *TCF7L2*), cell cycle regulation (*RB1*, *CDKN2A*), immune pathways (*B2M*, *HLA-A*), TGF-β superfamily signaling (*BMPR1A*), and receptor tyrosine kinase networks (*ERBB3*). However, this pattern did not persist in the MSS-restricted analysis and likely reflect the known prevalence of several of these genes within the MSI-high background. In contrast, *ERBB2/HER2*-amplified tumors represent molecularly discrete events with a sparse driver interaction profile. They demonstrate selective mutual exclusivity with *KRAS* and co-occurrence with *TP53*, consistent with a chromosomally unstable, *KRAS*-independent route of MAPK pathway activation. Hierarchical clustering further reinforced these distinctions across both anatomical sites and regardless of MSI status.

Our observed frequencies of *ERBB2/HER2* putative driver mutations (2.2%) and amplifications (2%) are consistent with previous large-scale studies reporting *ERBB2/HER2* mutations in 2-3% of CRCs and amplifications in 1.3-6% of cases, with some studies documenting combined *ERBB2/HER2* alterations in up to 7% of patients.^13^^,^^48–50^ Additionally, the observed age and sex distribution differences are consistent with previous studies in *ERBB2/HER2*-positive colorectal cancer, which have reported median ages ranging from 54 to 56 years and male predominance.^51^^,^^52^

As a landmark novel finding, our study revealed that, in contrast to *ERBB2/HER2*-amplified tumors, *ERBB2/HER2*-mutated CRCs are highly enriched with *KRAS* driver alterations (46.9%), unlike the study by Loree et al., in which *KRAS* mutations were noted to be approximately 25-35% in their institutional cohorts.^47^ The high rates of concurrent *KRAS* mutation noted in our study suggest that the “addiction” of *ERBB2/HER2*-mutated CRC cells to *ERBB2/HER2* activation might be limited by the requirement of a secondary oncogenic driver mutation of its own downstream signaling. Interestingly, *ERBB2/HER2*-altered tumors showed a depleted phenotype of concurrent *BRAF* V600E driver alterations, which is also notably different from previously published data.^47^ Targeting strategies directed solely at pathogenic *ERBB2/HER2* mutations might potentially result in a relatively modest benefit or even treatment failure, as the tumor cells might be capable of forming bypass loops in the MAPK pathway. Thereby, *ERBB2/HER2* mutations appear to be challenging targets due to high incidence rates. RAS oncogene mutations likely will require consideration of targeted approaches for RAS and HER2 oncogenes concurrently. Notably, the HER2-specific tyrosine kinase inhibitors have shown activity in *ERBB2/HER2*-mutated cancers, including breast and biliary cancers,^53^ and targetability of *ERBB2/HER2*-mutated RAS WT CRC can be addressed in similar basket trials in the future. It is also important to note that whether *ERBB2/HER2* mutations may in fact confer resistance to *KRAS*- and *BRAF*-directed precision therapies remains an important unanswered question that warrants further investigations to better define their predictive role.

Our finding that 37.2% of *ERBB2/HER2*-mutated tumors exhibit MSI aligns with recent reports showing strong associations between *ERBB2/HER2* mutations and hypermutated tumor phenotypes across gastrointestinal cancers.^54^ In contrast, the near absence of MSI in *ERBB2/HER2*-amplified tumors (1.6%) suggests these alterations arise through distinct mutational processes consistent with the chromosomal instability pathway that characterizes the majority of CRCs.^55^ The anatomical distribution patterns we observed, with *ERBB2/HER2*-mutated tumors enriched in the right colon and *ERBB2/HER2*-amplified tumors predominating in the left colon, align with previous reports demonstrating that *ERBB2/HER2* amplification occurs more frequently in left-sided and rectal tumors.^10^^,^^56^ These patterns parallel the established molecular distinctions between right- and left-sided CRCs. Right-sided tumors are enriched for CMS1 and CMS3 subtypes, while left-sided tumors predominantly exhibit CMS2 and CMS4 patterns.^4^^,^^57^

The co-occurrence analysis revealed distinct pathway interaction patterns that provide insights into potential therapeutic vulnerabilities. Although the co-occurrence patterns observed in overall *ERBB2/HER2*-mutated tumors were not demonstrated in the MSS-restricted cohort, likely due to reduced cohort size, the overall genomic landscape of this subgroup still carries potential biological and therapeutic implications worth considering. For example, *ARID1A* has been linked to resistance to anti-EGFR therapies in CRC.^58^ The *ERBB3* association may be more directly relevant to *ERBB2/HER2* biology, given that *ERBB3* serves as the preferred heterodimerization partner for *ERBB2/HER2*.^48^  *B2M* and *HLA-A* co-alterations are consistent with the MSI background and may reflect altered antigen-presenting mechanisms that could influence responses to anti-PD-1 therapy.^44^

In contrast, *ERBB2/HER2*-amplified tumors demonstrated a genomically sparse interaction profile, with only 2 significant associations: relative mutual exclusivity with *KRAS* and co-occurrence with *TP53*. The *KRAS* mutual exclusivity aligns with established patterns in CRC, where *ERBB2/HER2* amplification might serve as an alternative mechanism of MAPK pathway activation. The *TP53* co-occurrence, together with the higher fraction of genome altered observed in this subgroup, is in line with the chromosomal instability pathway of colorectal tumorigenesis. Early *TP53* loss enables the tolerance of widespread copy number alterations, including *ERBB2/HER2* amplification.^55^ Collectively, our findings suggest that *ERBB2/HER2*-targeted therapeutic strategies should be tailored according to the specific type of alteration. Mutations are associated with a multi-pathway dysregulated phenotype possibly requiring comprehensive targeted approaches with extensive potential resistance mechanisms, while amplifications could benefit from focused *ERBB2/HER2* inhibition in a *KRAS* wild-type context.

There are several limitations to our study, including the inherent limitations of the retrospective nature of the study; potential selection bias due to preexisting CRC datasets in the cBioPortal with specific inclusion/exclusion criteria; the manual composition of the 94-gene panel, and the absence of detailed clinical and demographic information, treatment history, and treatment-related survival outcomes. Notably, the heterogeneous nature of the aggregated datasets precluded meaningful analysis of clinical outcomes, therapeutic responses to HER2-targeted agents or tyrosine kinase inhibitors, and survival endpoints. Prospective studies specifically designed to capture these endpoints in *ERBB2/HER2* mutated vs amplified CRC populations are warranted. Additionally, *ERBB2/HER2* copy number alteration data were derived from heterogeneous genomic profiling platforms across contributing studies, which may introduce variability in amplification calls. Finally, MSS-restricted subgroup analyses, including hierarchical clustering and co-occurrence and mutual exclusivity patterns, were limited by reduced sample sizes within *ERBB2/HER2*-mutated and amplified subgroups, which may have reduced statistical power to detect associations observed in the overall cohort. The major strengths of our study include the novelty of the scientific questions investigated, the large cohort size curated from cBioPortal, and the ability to evaluate comprehensive molecular data that encompass multi-institutional genomic datasets.

## Conclusion

Our large-scale genomic profiling suggests that *ERBB2/HER2* alterations in colorectal cancer might encompass 2 biologically distinct entities. *ERBB2/HER2*-mutated tumors represent a distinct subgroup of CRC characterized by an MSI-enriched, hypermutated background with frequent concurrent *KRAS* driver alterations that may carry implications for resistance and may require combination targeted therapy approaches including RAS inhibitors, due to extensive co-mutation status. In contrast, *ERBB2/HER2*-amplified tumors exhibit chromosomally unstable patterns with depleted *KRAS* and *BRAF* driver alterations, *TP53* co-occurrence, and otherwise limited driver co-alterations, which may make them more amenable to focused *ERBB2/HER2*-targeted therapy. These exploratory findings highlight potentially different biological backgrounds of *ERBB2/HER2*-mutated and *ERBB2/HER2*-amplified CRC. Prospective studies are essential to validate our findings in these pathway interactions and evaluate treatment responses to targeted combinations.

## Supplementary Material

oyag302_Supplementary_Data

## Data Availability

All data used in this study are publicly available through cBioPortal for Cancer Genomics (https://www.cbioportal.org). Specific datasets and studies analyzed are referenced within the manuscript and can be accessed directly via the cBioPortal website.
