# Supplementary material for: Genomic landscape and clinical characteristics of ERBB2/HER2-mutated colorectal cancers
Source: Oncologist. 2026 Jul 31;31(9):oyag302. doi: 10.1093/oncolo/oyag302 (PMC13466721; doi:10.1093/oncolo/oyag302)
Supplement: Supplementary file 1 [file oyag302_supplementary_data.zip › Supplementary Figures Legends.docx]

**Supplementary Figure 1. Comprehensive genomic landscape of *ERBB2/HER2* and pathway alterations in the overall cohort of colorectal cancer (n=4,348).** Alteration frequencies are shown for 95 cancer-associated genes, including *ERBB2/HER2*, organized into eight major oncogenic pathways relevant to CRC biology: CRC core drivers/WNT signaling, RAS/RAF/MAPK signaling, PI3K/AKT/mTOR signaling, TGF-β/SMAD signaling, BMP signaling (SMAD-dependent), receptor tyrosine kinases, immune regulation/antigen presentation, and cell cycle/growth control. Within each pathway, genes are ordered by descending alteration frequency. For each gene, the alteration frequency represents the proportion of profiled patients harboring any genomic alteration. Patients without profiling data for a given gene were excluded from its frequency calculation.

**Supplementary Figure 2.** **Comprehensive genomic landscape of *ERBB2/HER2* and pathway alterations in colorectal cancer among microsatellite stable (MSS) patients (n=2,772)**. Alteration frequencies are shown for 95 cancer-associated genes, including *ERBB2/HER2*, organized into eight major oncogenic pathways relevant to CRC biology: CRC core drivers/WNT signaling, RAS/RAF/MAPK signaling, PI3K/AKT/mTOR signaling, TGF-β/SMAD signaling, BMP signaling (SMAD-dependent), receptor tyrosine kinases, immune regulation/antigen presentation, and cell cycle/growth control. Within each pathway, genes are ordered by descending alteration frequency. For each gene, the alteration frequency represents the proportion of profiled patients harboring any genomic alteration. Patients without profiling data for a given gene were excluded from its frequency calculation.

**Supplementary Figure 3. Comparative analysis of driver gene alterations across *ERBB2/HER2*-defined colorectal cancer subgroups in the overall cohort.** Putative driver alteration frequencies for selected key genes are shown across *ERBB2/HER2*-mutated (purple), *ERBB2/HER2*-amplified (green), and *ERBB2/HER2* wild-type (gray) subgroups. Genes are organized by pathway: CRC core/WNT signaling (*APC*, *TP53*, *RNF43*, *AXIN2*), RAS/RAF/MAPK signaling (*KRAS*, *BRAF* (all alterations), *BRAF* V600E, *NRAS*, *MAP2K1/2* & *MAPK1/3*), and PI3K/AKT/mTOR signaling (*PTEN*, *PIK3CA*). Statistical comparisons were performed using Fisher's exact test for overall group differences across all three subgroups, followed by pairwise comparisons with Benjamini-Hochberg false discovery rate correction applied across all pairwise tests. Significance levels indicate Benjamini-Hochberg-adjusted *p* values: **p* < 0.05, ***p* < 0.01, ****p* < 0.001.

**Supplementary Figure 4. Anatomical site-specific genomic clustering patterns in *ERBB2/HER2*-defined colorectal cancer subgroups among microsatellite stable (MSS) patients.** Unsupervised hierarchical clustering of genetic alteration frequencies across the 94-gene panel was performed separately for MSS colon cancers (left panel) and MSS rectal cancers (right panel) within *ERBB2/HER2*-mutated, amplified, and wild-type subgroups. Clustering was based on a subgroup-level gene alteration frequency matrix representing the percentage of patients harboring each alteration per subgroup, using Euclidean distance and complete linkage for both row and column clustering. Each row represents an individual gene, and the heatmap color intensity indicates alteration frequency. Dendrogram branches illustrate genomic similarity between *ERBB2/HER2* subgroups. This descriptive analysis demonstrates that across both anatomical sites, *ERBB2/HER2*-mutated and amplified tumors consistently occupy distinct clusters.

**Supplementary Figure 5. Anatomical site-specific genomic clustering patterns in *ERBB2/HER2*-defined colorectal cancer subgroups in the overall cohort.** Unsupervised hierarchical clustering of genetic alteration frequencies across the 94-gene panel was performed separately for colon cancers (left panel) and rectal cancers (right panel) within *ERBB2/HER2*-mutated, amplified, and wild-type subgroups. Clustering was based on a subgroup-level gene alteration frequency matrix representing the percentage of patients harboring each alteration per subgroup, using Euclidean distance and complete linkage for both row and column clustering. Each row represents an individual gene, and the heatmap color intensity indicates alteration frequency. Dendrogram branches illustrate genomic similarity between *ERBB2/HER2* subgroups. This descriptive analysis demonstrates that in both colon and rectal cancers, *ERBB2/HER2*-mutated tumors cluster separately from wild-type and amplified tumors.
