# Supplementary material for: Genomic landscape and clinical characteristics of ERBB2/HER2-mutated colorectal cancers
Source: Oncologist. 2026 Jul 31;31(9):oyag302. doi: 10.1093/oncolo/oyag302 (PMC13466721; doi:10.1093/oncolo/oyag302)
Supplement: Supplementary file 1 [file oyag302_supplementary_data.zip › sup fig 1.pdf]

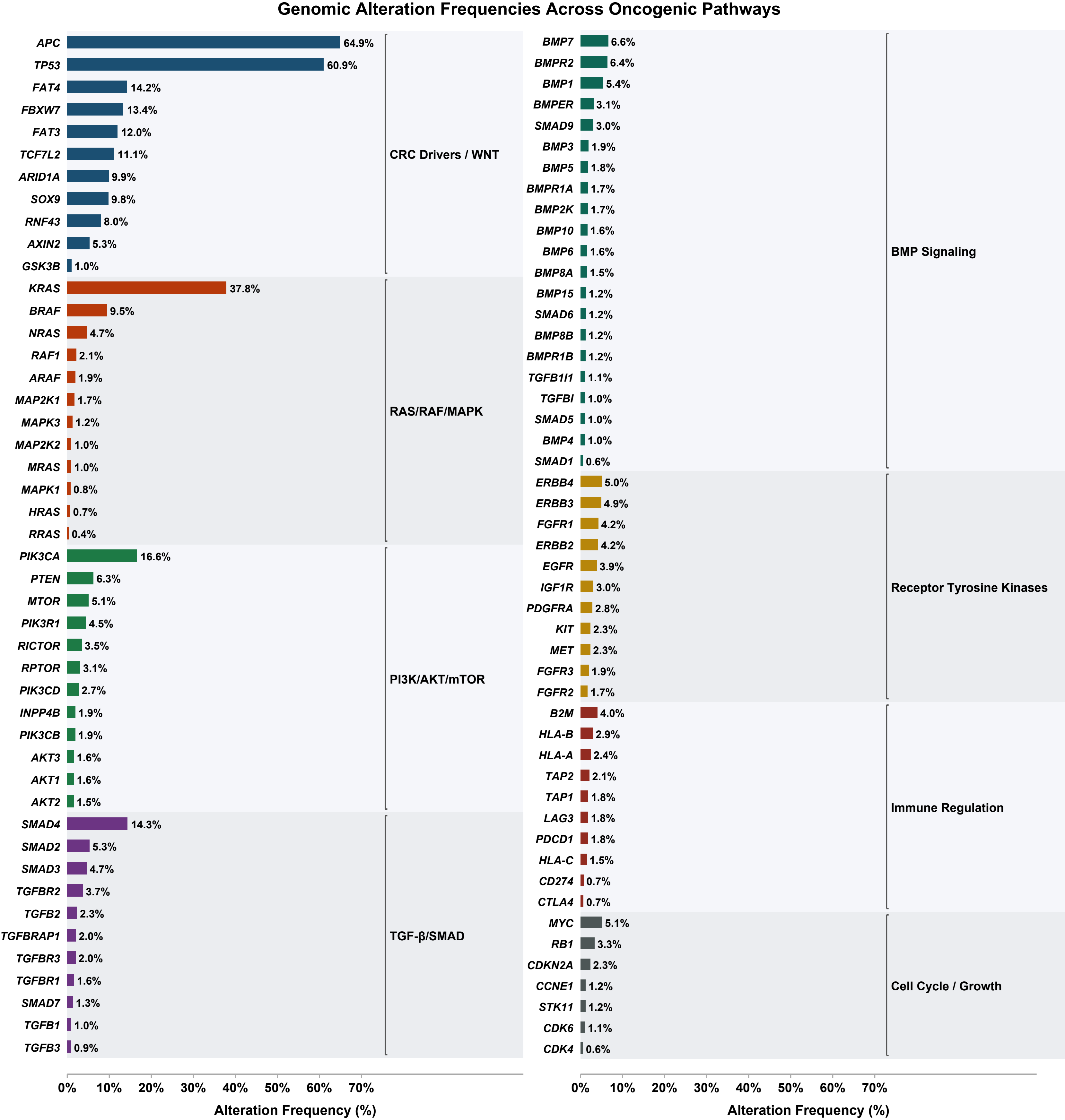
Genomic Alteration Frequencies Across Oncogenic Pathways
APC 64.9%
TP53 60.9%
FAT4 14.2%
FBXW7 13.4%
FAT3 12.0%
TCF7L2 11.1%
ARID1A 9.9%
SOX9 9.8%
RNF43 8.0%
AXIN2 5.3%
GSK3B 1.0%
CRC Drivers / WNT
KRAS 37.8%
BRAF 9.5%
NRAS 4.7%
RAF1 2.1%
ARAF 1.9%
MAP2K1 1.7%
MAPK3 1.2%
MAP2K2 1.0%
MRAS 1.0%
MAPK1 0.8%
HRAS 0.7%
RRAS 0.4%
RAS/RAF/MAPK
PIK3CA 16.6%
PTEN 6.3%
MTOR 5.1%
PIK3R1 4.5%
RICTOR 3.5%
RPTOR 3.1%
PIK3CD 2.7%
INPP4B 1.9%
PIK3CB 1.9%
AKT3 1.6%
AKT1 1.6%
AKT2 1.5%
PI3K/AKT/mTOR
SMAD4 14.3%
SMAD2 5.3%
SMAD3 4.7%
TGFBR2 3.7%
TGFB2 2.3%
TGFBRAP1 2.0%
TGFBR3 2.0%
TGFBR1 1.6%
SMAD7 1.3%
TGFB1 1.0%
TGFB3 0.9%
TGF-β/SMAD
BMP7 6.6%
BMPR2 6.4%
BMP1 5.4%
BMPER 3.1%
SMAD9 3.0%
BMP3 1.9%
BMP5 1.8%
BMPR1A 1.7%
BMP2K 1.7%
BMP10 1.6%
BMP6 1.6%
BMP8A 1.5%
BMP15 1.2%
SMAD6 1.2%
BMP8B 1.2%
BMPR1B 1.2%
TGFB1I1 1.1%
TGFBI 1.0%
SMAD5 1.0%
BMP4 1.0%
SMAD1 0.6%
BMP Signaling
ERBB4 5.0%
ERBB3 4.9%
FGFR1 4.2%
ERBB2 4.2%
EGFR 3.9%
IGF1R 3.0%
PDGFRA 2.8%
KIT 2.3%
MET 2.3%
FGFR3 1.9%
FGFR2 1.7%
Receptor Tyrosine Kinases
B2M 4.0%
HLA-B 2.9%
HLA-A 2.4%
TAP2 2.1%
TAP1 1.8%
LAG3 1.8%
PDCD1 1.8%
HLA-C 1.5%
CD274 0.7%
CTLA4 0.7%
Immune Regulation
MYC 5.1%
RB1 3.3%
CDKN2A 2.3%
CCNE1 1.2%
STK11 1.2%
CDK6 1.1%
CDK4 0.6%
Cell Cycle / Growth
0% 10% 20% 30% 40% 50% 60% 70%
Alteration Frequency (%)
0% 10% 20% 30% 40% 50% 60% 70%
Alteration Frequency (%)
