# Supplementary material for: Genomic landscape and clinical characteristics of ERBB2/HER2-mutated colorectal cancers
Source: Oncologist. 2026 Jul 31;31(9):oyag302. doi: 10.1093/oncolo/oyag302 (PMC13466721; doi:10.1093/oncolo/oyag302)

Genomic Alteration Frequencies Across Oncogenic Pathways in MSS Only

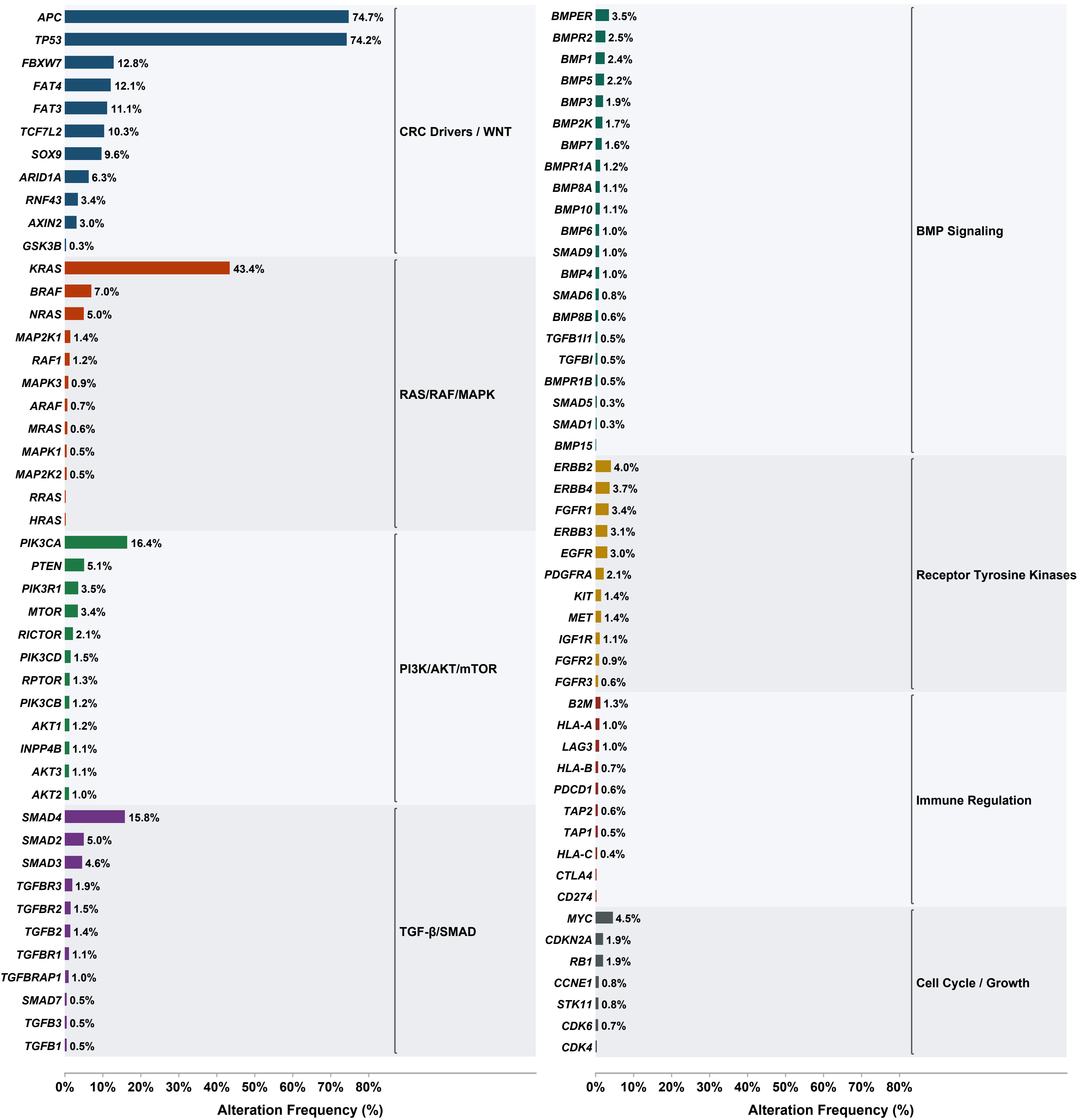

Supplement: Supplementary file 1 [file oyag302_supplementary_data.zip › sup fig 2.pdf]
