# Supplementary material for: Genomic landscape and clinical characteristics of ERBB2/HER2-mutated colorectal cancers
Source: Oncologist. 2026 Jul 31;31(9):oyag302. doi: 10.1093/oncolo/oyag302 (PMC13466721; doi:10.1093/oncolo/oyag302)
Supplement: Supplementary file 1 [file oyag302_supplementary_data.zip › supp fig 3.pdf]

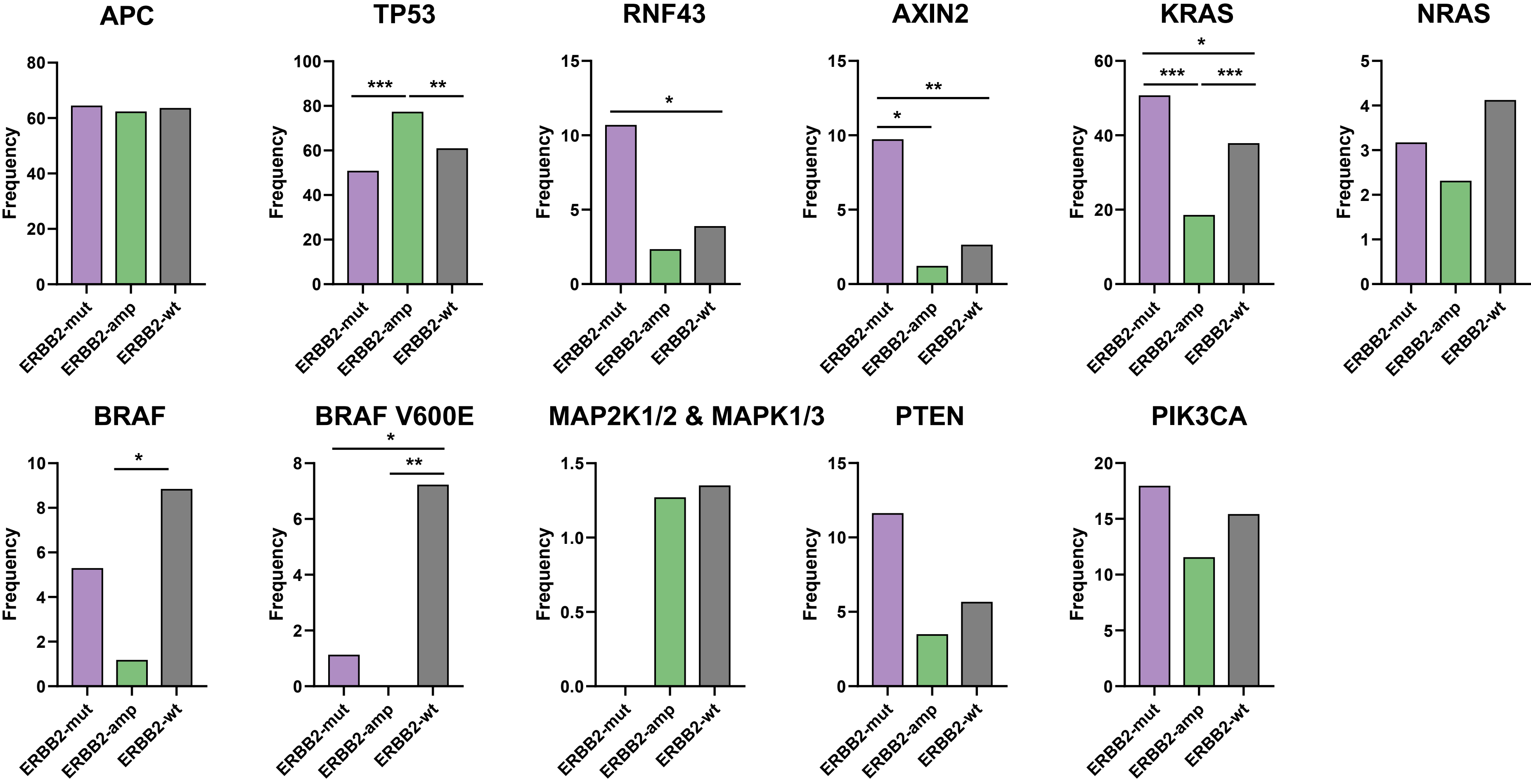
APC
TP53
RNF43
AXIN2
KRAS
NRAS
BRAF
BRAF V600E
MAP2K1/2 & MAPK1/3
PTEN
PIK3CA
Frequency
ERBB2-mut
ERBB2-amp
ERBB2-wt
*
**
***
