# Supplementary material for: Genomic landscape and clinical characteristics of ERBB2/HER2-mutated colorectal cancers
Source: Oncologist. 2026 Jul 31;31(9):oyag302. doi: 10.1093/oncolo/oyag302 (PMC13466721; doi:10.1093/oncolo/oyag302)
Supplement: Supplementary file 1 [file oyag302_supplementary_data.zip › supp fig 4.pdf]

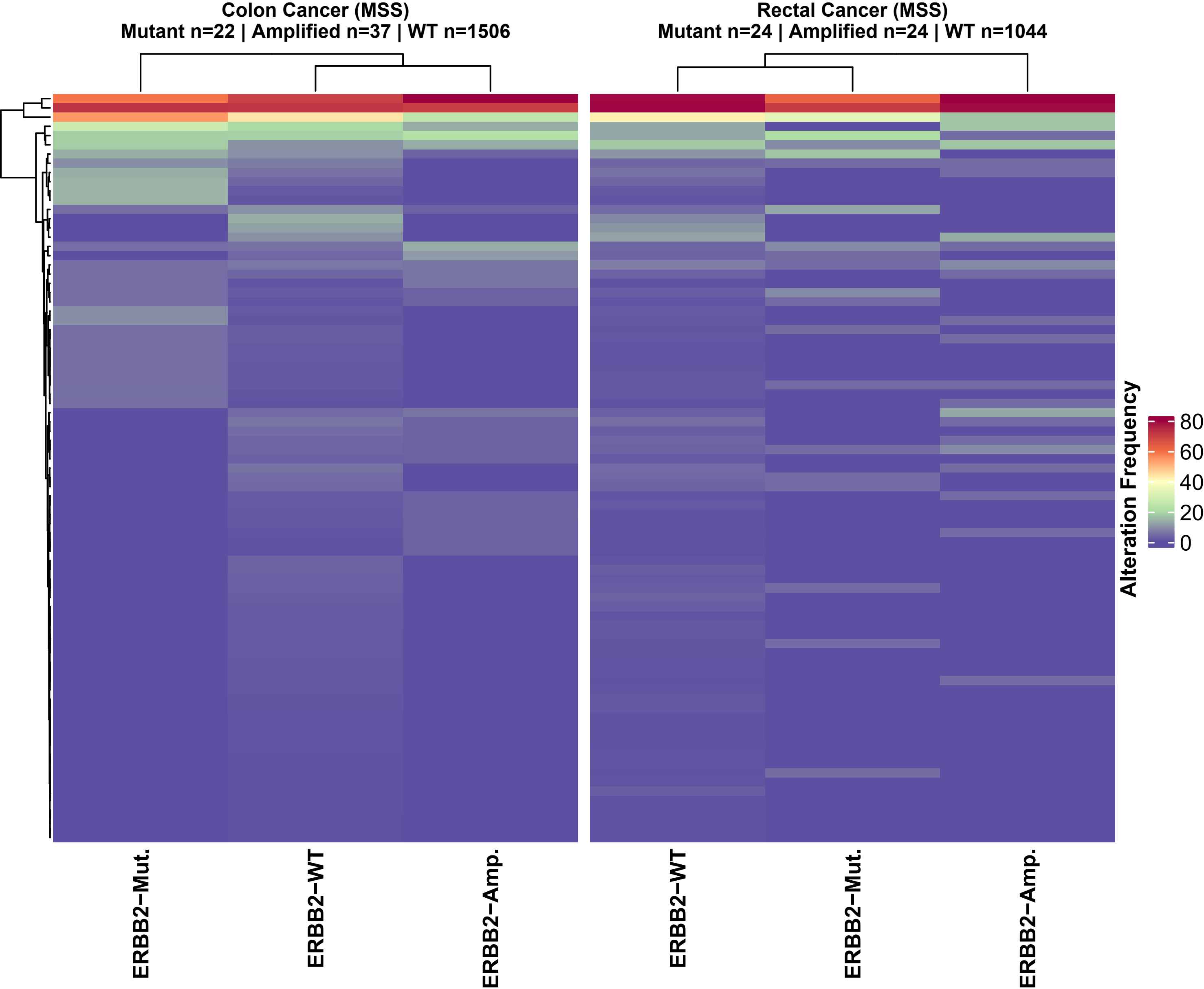
Colon Cancer (MSS)
Mutant n=22 | Amplified n=37 | WT n=1506
Rectal Cancer (MSS)
Mutant n=24 | Amplified n=24 | WT n=1044
ERBB2−Mut.
ERBB2−WT
ERBB2−Amp.
ERBB2−WT
ERBB2−Mut.
ERBB2−Amp.
Alteration Frequency
80
60
40
20
0
